# Phagocyte activity reflects mammalian homeo- and hetero-thermic physiological states

**DOI:** 10.1186/s12917-020-02450-z

**Published:** 2020-07-06

**Authors:** Jiri Pikula, Tomas Heger, Hana Bandouchova, Veronika Kovacova, Monika Nemcova, Ivana Papezikova, Vladimir Piacek, Renata Zajíčková, Jan Zukal

**Affiliations:** 1 Department of Ecology and Diseases of Zoo Animals, Game, Fish and Bees, University of Veterinary and Pharmaceutical Sciences Brno, Palackého třída 1946/1, 612 42 Brno, Czech Republic; 2 CEITEC - Central European Institute of Technology, University of Veterinary and Pharmaceutical Sciences Brno, Brno, Czech Republic; 3 Department of Botany and Zoology, Masaryk University, Kotlářská 2, 611 37 Brno, Czech Republic; 4 Institute of Biostatistics and Analyses, Masaryk University, Kamenice 3, 625 00 Brno, Czech Republic; 5 Institute of Vertebrate Biology, Czech Academy of Sciences, Květná 8, 603 65 Brno, Czech Republic

**Keywords:** Bats, Torpor, Innate immunity, Blood, Phagocytosis, Respiratory burst

## Abstract

**Background:**

Emergence of both viral zoonoses from bats and diseases that threaten bat populations has highlighted the necessity for greater insights into the functioning of the bat immune system. Particularly when considering hibernating temperate bat species, it is important to understand the seasonal dynamics associated with immune response. Body temperature is one of the factors that modulates immune functions and defence mechanisms against pathogenic agents in vertebrates. To better understand innate immunity mediated by phagocytes in bats, we measured respiratory burst and haematology and blood chemistry parameters in heterothermic greater mouse-eared bats (*Myotis myotis*) and noctules (*Nyctalus noctula*) and homeothermic laboratory mice (*Mus musculus*).

**Results:**

Bats displayed similar electrolyte levels and time-related parameters of phagocyte activity, but differed in blood profile parameters related to metabolism and red blood cell count. Greater mouse-eared bats differed from mice in all phagocyte activity parameters and had the lowest phagocytic activity overall, while noctules had the same quantitative phagocytic values as mice. Homeothermic mice were clustered separately in a high phagocyte activity group, while both heterothermic bat species were mixed in two lower phagocyte activity clusters. Stepwise regression identified glucose, white blood cell count, haemoglobin, total dissolved carbon dioxide and chloride variables as the best predictors of phagocyte activity. White blood cell counts, representing phagocyte numbers available for respiratory burst, were the best predictors of both time-related and quantitative parameters of phagocyte activity. Haemoglobin, as a proxy variable for oxygen available for uptake by phagocytes, was important for the onset of phagocytosis.

**Conclusions:**

Our comparative data indicate that phagocyte activity reflects the physiological state and blood metabolic and cellular characteristics of homeothermic and heterothermic mammals. However, further studies elucidating trade-offs between immune defence, seasonal lifestyle physiology, hibernation behaviour, roosting ecology and geographic patterns of immunity of heterothermic bat species will be necessary. An improved understanding of bat immune responses will have positive ramifications for wildlife and conservation medicine.

## Background

Phagocytosis is a protective mechanism linking innate and adaptive immune responses [1, 2]. The primary function of phagocytes is to recognise, engulf and destroy pathogenic agents, infected or dead cells and foreign particles. For this purpose, phagocytes are recruited to sites of infection, tissue damage or irritation by foreign particles. In order to produce potent microbicidal agents, such as reactive oxygen and nitrogen species (ROS, RNS), phagocytes gain energy from the catabolism of glucose and thereby considerably increase the uptake of oxygen [2]. In addition to their fundamental function in the innate immune system, ROS and RNS also modulate adaptive immune responses. While techniques to measure phagocyte activity through respiratory burst have relevance for clinical medicine [2, 3], they can also be employed in comparative and ecological immunology [4, 5].

Phagocytic cells of the mammalian immune system include monocytes and macrophages, dendritic cells, neutrophils and other granulocytes [1]. A host’s ability to defend against pathogens can be evaluated using either separate cell types or all phagocytes present in whole blood. With the latter option, micro-method procedures allow for the examination of small wildlife species [6]. The magnitude of respiratory burst is enhanced by priming of phagocytes by previous exposure to microorganisms and depends on the total and differential count of cells [3, 6].

Another important factor modulating immune function is temperature. In homeotherms, the costs associated with mounting an immune response are traded against thermoregulation [7, 8], while phagocyte activity varies with conditions associated with thermal acclimatisation in poikilotherms [4]. Likewise, expression of both daily heterothermy [9] and winter torpor/arousal bouts [10] have profound effects on immune defence.

Some highly pathogenic zoonotic agents circulate in bats without causing severe infections in these reservoir hosts [11]. On the other hand, temperate bat species populations are threatened by a fungal skin disease [12]. Greater insights into bat immune defence mechanisms are therefore needed.

In general, the annual cycle of temperate insectivorous bat species comprises 1) a period of food availability when active bats maintain homeothermy, and 2) a winter period when bats minimise their metabolic needs through torpor [13]. Hibernation temperatures in temperate vespertilionid and rhinolophid bats range from 0 to 12 °C [14, 15] and, during torpor, the bats lower their body temperature close to the ambient temperature, together with reductions in metabolic, heart and respiratory rates [16]. Bats have adapted to profound changes in their metabolism, oxygen consumption and other physiological functions at the organismal, organ, cellular and molecular levels to cope with the cycles of torpor and arousal [17–19]. Regulation of body temperature is not that simple in temperate bats, however, and bats are also known to use daily torpor in order to save energy and water at cold ambient temperatures and during periods of food scarcity in their active and/or reproductive seasons [20, 21]. Moreover, in addition to rewarming periodically to euthermy, hibernating bats may also display heterothermic, and even cold arousals [16, 22]. Irregular periodicity in the torpor/arousal cycle means that bats will be in different physiological states when undergoing prolonged torpor, rewarming from torpor, euthermy or cooling into torpor [16]. Physiological characteristics associated with the role of low hibernation body temperature in phagocyte respiratory burst response include 1) a decrease in the level of blood glucose [23, 24]; 2) lowered oxygen availability for phagocyte respiratory burst due to lowered venous and arterial blood partial pressure of oxygen, a higher oxygen affinity to haemoglobin [25], and long periods of apnoe [26]; 3) torpor induced leukopenia/neutropenia [27, 28]; and 4) altered activity of complement factors [29, 30].

In light of the above, heterotherms provide an interesting model for examining immune system function. Given that many parameters impacting phagocyte respiratory burst are altered during torpor, we predict lowered phagocyte respiratory burst performance in heterothermic animals compared with homeotherms. We performed a functional comparative study to confirm this by sampling blood from heterothermic greater mouse-eared bats (*Myotis myotis*) and noctules (*Nyctalus noctula*) and homeothermic laboratory mice (*Mus musculus*), evaluating phagocyte activity in combination with standard haematology and blood chemistry as predictors of both time-related and quantitative parameters of respiratory burst.

## Results

**Fig. 1:**
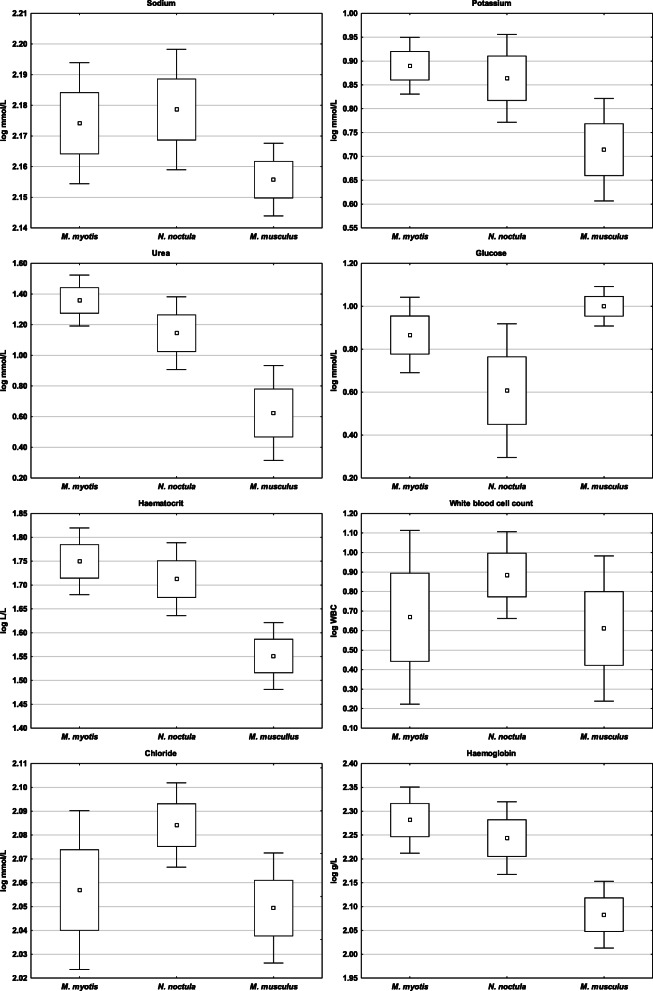
Differences in blood profile parameters measured in homeothermic mice and heterothermic bats. Blood collection was performed from *Myotis myotis* and *Nyctalus noctula* bats and laboratory mice at body temperatures of 25 °C and 38 °C reflecting their respective physiological states

**Fig. 2:**
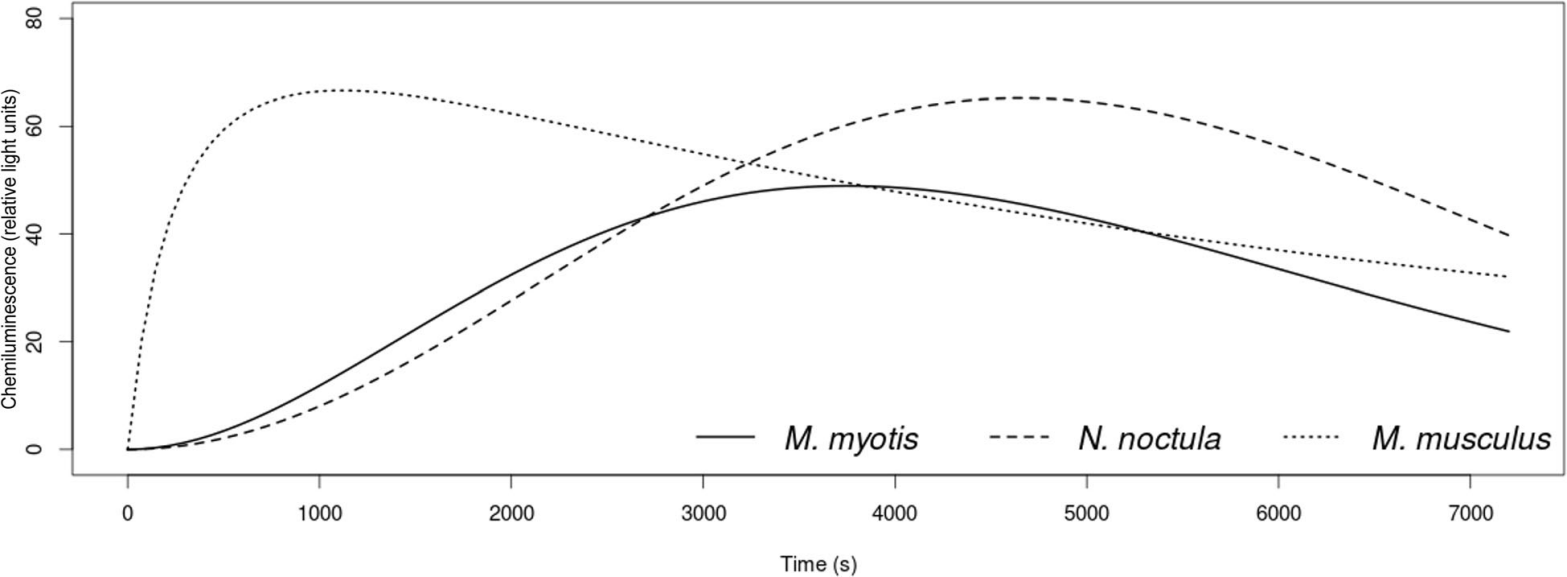
Phagocyte activity kinetics in homeothermic mice and heterothermic bats. Chemiluminescence represents the intensity of respiratory burst of phagocytes over time. Luminometric detection was performed at cell incubation temperatures of 25 °C for *Myotis myotis* and *Nyctalus noctula* bats and 38 °C for laboratory mice, reflecting their physiological state based on body temperature measurements at the time of blood collection

**Fig. 3:**
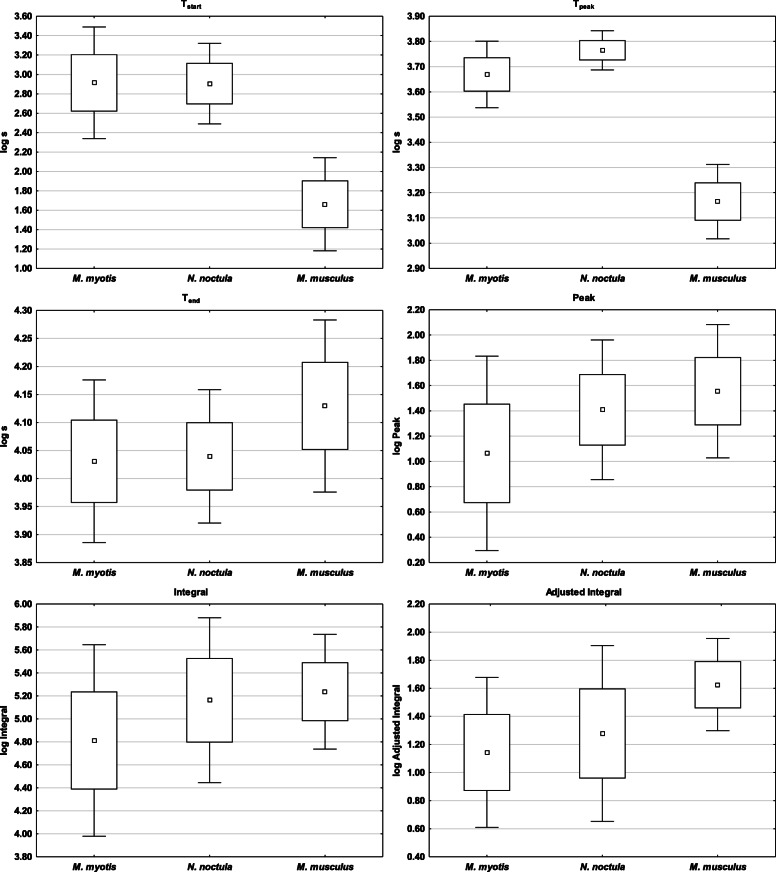
Differences in phagocyte respiratory burst parameters measured in homeothermic mice and heterothermic bats. Legend: T_start_ = time-to-start of phagocyte respiratory burst response, T_peak_ = time-to-peak of phagocyte respiratory burst response, T_end_ = time-to-end of phagocyte respiratory burst response, Peak = peak intensity of phagocyte respiratory burst, Integral = total phagocyte capacity, Adjusted Integral = total phagocyte capacity recalculated for the white blood cell count

With the exception of total dissolved carbon dioxide (tCO_2_) and total phagocyte capacity (Integral), the three mammal species in this study differed in all blood profile and phagocyte respiratory burst parameters (Table 1), with post-hoc comparisons confirming species-specific differences in different parameters. The two bat species had similar electrolyte levels (Na, K) and time-related parameters of phagocyte activity (T_start_, T_end_), but differed in blood profile parameters related to metabolism (Urea, Glu) and red blood cell count (Hct, Hb) (Figs. 1 and 2, Table 1). While heterothermic *N. noctula* bats had the same quantitative values (Peak, Integral) as laboratory mice, heterothermic *M. myotis* differed in all phagocyte activity parameters (Figs. 2 and 3, Table 1). Nevertheless, k-means clustering analysis clearly separated all laboratory mice specimens from bats at the highest Euclidean distance (0.382 “high phagocytic activity group, cluster 2” and 0.519 “low phagocytic activity group, cluster 3”, respectively). The splitting of bats into two additional clusters (2 and 3; Euclidean distance 0.279) was not driven by species specificity but mainly by parameters related to white blood cell count (WBC) and phagocyte respiratory burst (Table 2).

**Table 1:** Differences in blood profile and phagocyte activity parameters measured in greater mouse-eared bats (*Myotis myotis*), noctules (*Nyctalus noctula*) and laboratory BALB/C mice (*Mus musculus*). Post-hoc comparisons were used to evaluate species-specific differences in parameters. Legend: Hct = haematocrit, Hb = haemoglobin, Na = sodium, K = potassium, Cl = chloride, Urea = blood urea nitrogen, Glu = glucose, tCO_2_ = total dissolved carbon dioxide, WBC = white blood cell count, T_start_ = time-to-start of phagocyte respiratory burst response, T_peak_ = time-to-peak of phagocyte respiratory burst response, T_end_ = time-to-end of phagocyte respiratory burst response, Peak = peak intensity of phagocyte respiratory burst, Integral = total phagocyte capacity, Adjusted Integral = total phagocyte capacity recalculated for the white blood cell count; Mmyo = *Myotis myotis,* Nnoc = *Nyctalus noctula* and Mus = *Mus musculus*, ns = non-significant

Analysis of variance									LSD post-hoc comparisons			
Log-transformed variable	SS Model	df Model	MS Model	SS Residual	df Residual	MS Residual	F	p	Mmyo vs Nnoc	Mmyo vs Mus	Nnoc vs Mus	Remark
**Hct (L/L)**	0.27	2	0.14	0.04	33	0.00	101.31	**< 0.001**	0.021	< 0.001	< 0.001	Species-specific
**Hb (g/L)**	0.27	2	0.14	0.04	33	0.00	101.64	**< 0.001**	0.019	< 0.001	< 0.001	Species-specific
**Na (mmol/L)**	0.00	2	0.00	0.00	33	0.00	23.73	**< 0.001**	ns	< 0.001	< 0.001	Homeotherm differs from heterotherms
**K (mmol/L)**	0.22	2	0.11	0.07	33	0.00	52.81	**< 0.001**	ns	< 0.001	< 0.001	Homeotherm differs from heterotherms
**Cl (mmol/L)**	0.01	2	0.00	0.01	33	0.00	24.84	**< 0.001**	< 0.001	ns	< 0.001	Heterotherm species differ
**Urea (mmol/L)**	3.48	2	1.74	0.53	33	0.02	108.08	**< 0.001**	< 0.001	< 0.001	< 0.001	Species-specific
**Glu (mmol/L)**	0.99	2	0.49	0.38	33	0.01	42.41	**< 0.001**	< 0.001	0.005	< 0.001	Species-specific
**tCO**_**2**_**(mmol/L)**	0.01	2	0.00	0.13	32	0.00	1.16	0.327	ns	ns	ns	
**WBC**	0.51	2	0.25	1.09	33	0.03	7.72	**< 0.002**	0.007	ns	< 0.001	Heterotherm species differ
**T**_**start**_	12.92	2	6.46	2.08	33	0.06	102.69	**< 0.001**	ns	< 0.001	< 0.001	Homeotherm differs from heterotherms
**T**_**peak**_	2.60	2	1.30	0.13	33	0.00	328.98	**< 0.001**	< 0.001	< 0.001	< 0.001	Species-specific
**T**_**end**_	0.07	2	0.04	0.17	32	0.01	7.07	**0.003**	ns	0.002	0.005	Homeotherm differs from heterotherms
**Peak**	1.49	2	0.74	3.28	33	0.10	7.47	**0.002**	0.013	< 0.001	ns	Heterotherm species differ
**Integral**	1.19	2	0.60	4.06	33	0.12	4.84	0.014	0.022	0.006	ns	Heterotherm species differ
**Adjusted Integral**	1.52	2	0.76	2.20	33	0.07	11.36	**< 0.001**	ns	< 0.001	0.002	Homeotherm differs from heterotherms

**Table 2:** Mean values of blood profile and phagocyte activity parameters in groups defined by K-means clustering analysis of the three mammal species under study. All *Mus musculus* specimens separated from bats in one cluster. Bats were split between two additional groups with clustering not driven by species specificity but rather parameters related to white blood cell count and phagocyte respiratory burst. Legend: Hct = haematocrit, Hb = haemoglobin, Na = sodium, K = potassium, Cl = chloride, Urea = blood urea nitrogen, Glu = glucose, tCO_2_ = total dissolved carbon dioxide, WBC = white blood cell count, T_start_ = time-to-start of phagocyte respiratory burst response, T_peak_ = time-to-peak of phagocyte respiratory burst response, T_end_ = time-to-end of phagocyte respiratory burst response, Peak = peak intensity of phagocyte respiratory burst, Integral = total phagocyte capacity, Adjusted Integral = total phagocyte capacity recalculated for the white blood cell count; Mmyo = *Myotis myotis,* Nnoc = *Nyctalus noctula* and Mus = *Mus musculus*

	Cluster 1	Cluster 2	Cluster 3
**Hct (L/L)**	1.551	1.716	1.744
**Hb (g/L)**	2.083	2.248	2.276
**Na (mmol/L)**	2.155	2.179	2.172
**K (mmol/L)**	0.714	0.878	0.874
**Cl (mmol/L)**	2.049	2.079	2.062
**Urea (mmol/L)**	0.623	1.215	1.279
**Glu (mmol/L)**	0.999	0.665	0.802
**tCO**_**2**_**(mmol/L)**	1.374	1.351	1.342
**WBC**	0.610	0.876	0.677
**T**_**start**_	1.661	2.759	3.071
**T**_**peak**_	3.164	3.727	3.709
**T**_**end**_	4.129	4.078	3.991
**Peak**	1.555	1.526	0.936
**Integral**	5.236	5.310	4.650
**Adjusted Integral**	1.626	1.434	0.973
**Individuals in the cluster**	Mus1–13	Mmyo3,21–23 Nnoc1,4–7,9,10,12	Mmyo1,2,5 -7,10,12 Nnoc2,3,8,11

**Fig. 4:**
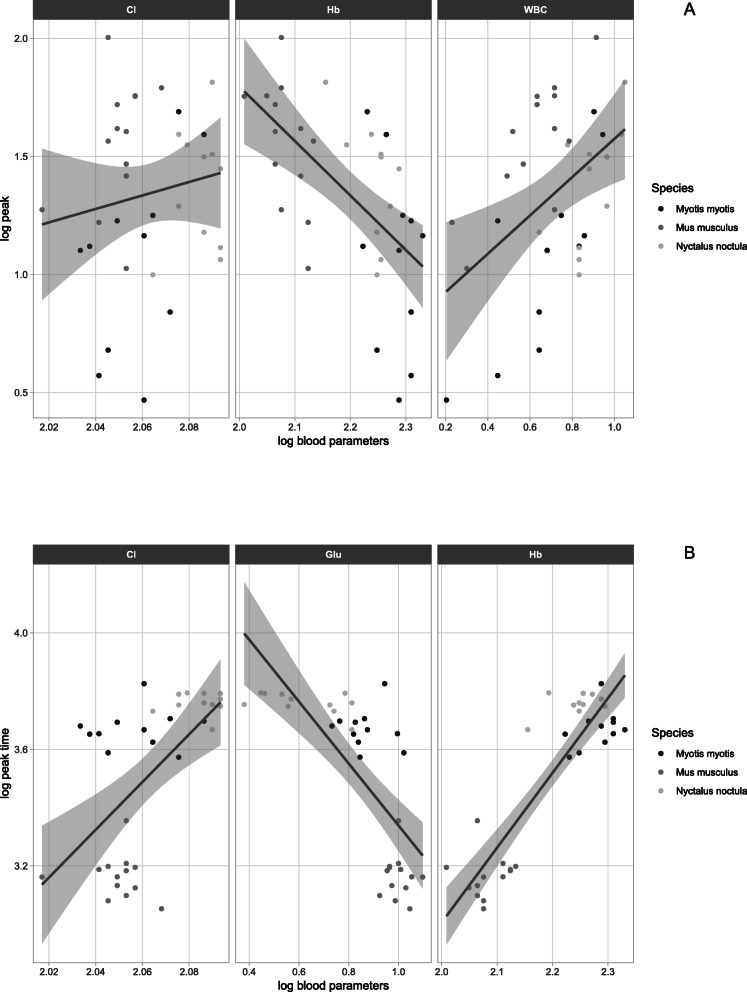
Predictors of phagocyte activity. Blood profile parameters relevant for quantitative Peak (**a**) and time-related T_peak_ (**b**) identified using stepwise regression. Legend: Hb = haemoglobin, Cl = chloride, Glu = glucose, WBC = white blood cell count, T_peak_ = time-to-peak of phagocyte respiratory burst response, Peak = peak intensity of phagocyte respiratory burst*.* Confidence intervals shown as shaded areas

All predictive regression models were statistically highly significant and, with the exception of variables T_end_ and Adjusted Integral (AI) that had lower coefficients of determination, there was a good fit with the data (Table 3). Forward and backward stepwise regression both showed differences in the factors explaining quantitative and time-related phagocyte activity parameters. Quantitative phagocyte activity parameters (Peak, Integral, Adjusted Integral) were positively correlated with white blood cell count and negatively with haemoglobin concentration (Table 3, Fig. 4a), while time-related parameters (T_start_, T_peak_) were positively influenced by haemoglobin concentration (Table 3, Fig. 4b). The time-to-end response (T_end_) differed from the other time-related parameters in being influenced by the same factors as the quantitative parameters.

**Table 3:** Stepwise multiple regression models predicting parameters of phagocyte activity in greater mouse-eared bats (*Myotis myotis*), noctules (*Nyctalus noctula*) and laboratory BALB/C mice (*Mus musculus*). Legend: Hb = haemoglobin, Cl = chloride, Glu = glucose, tCO_2_ = total dissolved carbon dioxide, WBC = white blood cell count, T_start_ = time-to-start of phagocyte respiratory burst response, T_peak_ = time-to-peak of phagocyte respiratory burst response, T_end_ = time-to-end of phagocyte respiratory burst response, Peak = peak intensity of phagocyte respiratory burst, Integral = total phagocyte capacity, Adjusted Integral = total phagocyte capacity recalculated for the white blood cell count; * = *p* < 0.05 (not significant after Bonferroni correction), ** = *p* < 0.01, *** = *p* < 0.001

Phagocyte activity parameters	Intercept	Predicting model parameters				Multiple R2
**Time-related**						
T_start_	−8.680***	−0.849 Glu*	5.531 Hb***	−0.419 WBC		0.87
T_peak_	−4.438	1.840 Cl	−0.360 Glu*	2.028 Hb***		0.88
T_end_	4.273***	0.370 tCO_2_*	0.121 Glu	−0.426 Hb**	0.184 WBC**	0.56
**Quantitative**						
Peak	−0.478	3.739 Cl	−2.972 Hb***	0.889 WBC***		0.76
Integral	9.774***	−2.505 Hb***	1.103 WBC***			0.70
Adjusted Integral	6.754***	−2.463 Hb***				0.58

## Discussion

Both functional competence of the host immune system and microbial virulence determine whether an infection becomes established or is cleared following interaction between the host and a pathogen [31]. As phagocyte respiratory burst is a crucial part of the host’s innate defence mechanism [2], it is supposed to work efficiently in the metabolic and cellular milieu of blood regulated by homeostatic mechanisms. However, microorganisms have evolved in parallel to the evolution of the host’s immune system, and display a variety of mechanisms for evading phagocytic destruction [32].

Here, we show a high-to-low gradient of differential phagocyte activity performance in homeotherms and heterotherms. While multivariate analysis clustered homeothermic laboratory mice in a high phagocyte activity group, both heterothermic bat species were mixed in two lower phagocyte activity clusters, though our data indicate that *N. noctula* were clustered closer to homeothermic laboratory mice, as manifested by similar quantitative values of phagocyte respiratory burst. We can only speculate that noctules require a higher phagocytic activity set point during torpor because they hibernate in roosts subject to more rapid temperature changes compared with the more stable cave environment utilised by *M. myotis* [33–35].

Despite significant multi- and uni-variate differences in blood profile and phagocyte respiratory burst parameters, it was possible to formulate general predictions based on the data from the homeothermic and heterothermic mammals examined, with stepwise regressions identifying relevant blood profile variables (i.e. glucose, white blood cell count, haemoglobin, total dissolved carbon dioxide and chloride) as predictors of phagocyte activity. While resting phagocytes are low in metabolism, activation of respiratory burst triggers oxygen and energy consumption [2]. In the present study, heterothermic bats displayed significantly lower blood glucose levels than the laboratory mice. Interestingly, a negative relationship between glucose and time-related phagocyte activity parameters was observed, but not statistically significant after *p*-value correction. This finding suggests either a low glucose level threshold for respiratory burst or a switch of fuel sources from carbohydrates to fatty acids in hypoglycaemic torpid bats [30].

White blood cell counts, representing phagocyte numbers available for respiratory burst, proved the best predictors of both time-related and quantitative parameters of phagocyte activity. Based on flow cytometry of rodent blood, Lojek et al. [6] concluded that the majority of whole blood respiratory burst is due to neutrophils, the most abundant and potent phagocytes and the first cells recruited to sites of infection [24, 36, 37]. During torpor, the numbers of all white blood cell types in circulation decrease considerably [27]; surprisingly, however, the homeothermic mice in this study displayed the lowest white blood cell count. While neutrophils are subject to margination in blood vessels during torpor, their numbers are usually restored rapidly as heterothermic individual re-warm upon arousal [28].

Blood haemoglobin concentration in this study was considered as a proxy variable for oxygen available for uptake by phagocytes [2]. Compared with the laboratory mice, both bat species displayed higher haematocrit and haemoglobin levels, suggesting an extraordinary oxygen-carrying capacity [25, 38, 39]. Haemoglobin concentration, as an indicator of oxygen availability, correlated with the onset of phagocytosis, i.e. the time-related respiratory burst parameters T_start_ and T_peak_ (Table 3, Fig. 4b); surprisingly, however, we recorded a negative relationship between haemoglobin and T_end_ and all quantitative phagocyte activity variables. This unexpected finding may have been due to an increased affinity of haemoglobin for oxygen at lower temperatures in torpid bats [25], the quenching effect of haemoglobin [40] and/or scavenging of hydrogen peroxide by catalase produced by red blood cells [41].

As a principal extracellular fluid electrolyte, the chloride anion is critical for maintaining water and acid-base balance, with the kidneys regulating blood chloride concentrations within a narrow range. Chloride also features in the respiratory burst reaction, where it is catalysed by phagocyte myeloperoxidase to generate chlorinating oxidants such as hypochlorous acid [42, 43]. Though not statistically significant, the chloride variable was included in the model predicting the phagocyte activity parameters T_peak_ and Peak. In bats infected by the white-nose syndrome fungus (*Pseudogymnoascus destructans*) and amphibians affected by chytridiomycosis, chloride loss through damaged skin may thus be a factor modulating phagocytic activity and reducing their innate defences [44, 45].

Important issues in this type of study are the blood collection technique employed and the time required for sample processing. While some haematology and serum chemistry parameters may alter in response to physical restraint during blood collection [46], stress associated with short-term capture and handling has little adverse effect on innate immune parameters [47]. Further, as blood cells continue to consume glucose and phagocytes can still be activated in the stored blood sample [6], we used an on-site analyser to measure blood profile parameters immediately following collection, with subsequent examination of phagocyte respiratory burst taking place in the laboratory within one hour. Hence, we are satisfied that our readings provide an accurate representation of the factors associated with phagocyte respiratory burst in homeothermic and heterothermic small mammals.

Both the duration and thermal profile of hibernation, as well as daily torpor utilisation, can have a significant influence on the activity of pathogenic agents [48–50]. It has been suggested that mammalian endothermy and homeothermy have evolved as trade-offs between metabolic costs and avoidance of the temperature preference ranges of many microorganisms [51]. The hibernation behaviour of bats infected with the psychrophilic white-nose syndrome fungus, for example, may modulate defence mechanism function in two ways: 1) they may show a fever response, meaning that they re-warm to a higher temperature during arousal [52] and 2) their choice of hibernation temperature could have an impact on fungal growth rate, infection intensity, pathophysiology and the ultimate outcome of the skin disease [24].

Phagocytes produce reactive oxidants to destroy pathogens; however, these can induce self-damage as well. Indeed, oxidative stress is frequently associated with pathogenesis of infections and immunopathology of chronic inflammation [53–55]. Bats are relatively unique in their ability to mitigate oxidative stress associated with increased oxidant generation during flight and immune response to intracellular infection [56, 57], with antioxidant mechanisms appearing to contribute to the bat’s reservoir status, allowing them to tolerate pathogens highly virulent to other mammalian species, including humans [11, 58]. Using an in vitro comparative experiment with *M. myotis* bone marrow-derived macrophages and laboratory mice, Kacprzyk et al. [59] demonstrated that bat cells, unlike murine cells, show a balanced pro- and anti-inflammatory response following artificial immunity challenge. As noted in Palearctic hibernating bat species infected with the white-nose syndrome fungus, longer-term co-evolution in regions of pathogen endemicity may also promote disease tolerance to extracellular agents [49, 60]. Unlike Palearctic bats, North American bats show a dysregulated immune response to white-nose syndrome early in the post-hibernation period [61]. The results of the present study suggest that pathogen tolerance in hibernating bats may be associated with decreased phagocyte respiratory burst rather than mitigation of adverse effects associated with phagocyte activation [62], with tolerance mechanisms associated with lower immune activity protecting the infected host from damage by both the pathogen and the immune system. As tolerance has little direct negative effect on the infectious agent, this promotes the reservoir status of bats, with consequences for host-pathogen co-evolution [63].

We sampled early post-hibernation *M. myotis* and *N. noctula* bats captured in their hibernacula for the comparison with the laboratory mouse as a standard immunological model matching both bat species in size and predicted that body temperature is the main driver of phagocyte activity. Our study design based on sampling bat species at different hibernacula without spatial replication, however, might confound comparative results. Bat species used in the study utilize different roosting strategies, meaning that they never meet in the same shelter type. It is clear that there is spatial variation in immune defences [64], however, both bat species are not sedentary to allow for easy inference of spatial immunity patterns. *Myotis myotis* bat is a regionally migrating species with regular movements associated with its annual life-cycle of about 150 km. Individuals sampled in a specific hibernaculum are aggregated there randomly from a large geographic region [65, 66]. Likewise, *Nyctalus noctula* is a migratory bat species with movements to hibernacula of 1000 km or even longer [67]. The measured immune responses of non-sedentary bat species thus probably reflect greater spatial variation. Lacking comprehensive knowledge about other (transitional and summer) roosting sites of sampled individuals and their exposure to factors of environmental variation and pathogen load [68, 69], it was impossible to adopt a study design to decipher the contribution of particular roosting ecology to the immune function.

## Conclusions

Emergence of both viral zoonoses from bats and diseases that threaten bat populations has highlighted the necessity for greater insights into the functioning of the bat immune system [12, 70–72]. Particularly when considering hibernating temperate bat species, it is important to understand the seasonal dynamics associated with immune response. In this study, we provide comparative functional data suggesting that phagocyte activity, as an essential mechanism of innate immunity, reflects the physiological state and blood metabolic and cellular characteristics of homeothermic and heterothermic mammals. Further studies will be necessary to elucidate trade-offs between immune competence, seasonal lifestyle physiology, hibernation behaviour, roosting ecology and geographic patterns of immunity in heterothermic bat species.

## Methods

### Blood sampling and measurement

We used bat heterotherms and laboratory mice for the comparative study of phagocyte respiratory burst. Each bat and mouse was handled in the least stressful manner possible by a trained veterinarian in order to obtain non-terminal samples. A total of 14 adult BALB/c (Bagg and Albino) laboratory mice of equal sex ratio were purchased from a commercial breeder (Velaz, Prague, Czech Republic). In a facility accredited for housing laboratory rodents (University of Veterinary and Pharmaceutical Sciences Brno, Czech Republic), mice were kept under standard conditions in boxes for rodents using wood shavings as bedding. Experimental mice were fed granules for rodents, a mixture of seeds, meadow grass and hay and were provided with drinking water ad libitum. Blood samples were collected from the saphenous vein of mice without anaesthesia [73]. During the early post-hibernation period (April), we sampled 11 specimens of *M. myotis*, a cave-dwelling hibernator, and 12 specimens of *N. noctula*, a rock-crevice-dwelling and/or tree-hole hibernator, i.e. two bat species with different hibernation behaviour. Wild *Myotis myotis* bats were netted emerging from the Sloupsko-Šošůvské caves [49.4104556 N, 16.7390147E]. *N. noctula* bats, rescued as a colony from a building in Ivanovice na Hané [49.3054183 N, 17.0934306E], were submitted to a wildlife rescue centre in the Department of Ecology and Diseases of Game, Fish and Bees, University of Veterinary and Pharmaceutical Sciences Brno, Czech Republic. After skin disinfection with alcohol, 120 μl blood samples were taken from bats by puncturing the uropatagial vessel using a sterile needle, the sample being collected using a heparinised pipette tip [24, 74]. The puncture site was then compressed and sealed with a drop of surgical tissue glue (Surgibond, SMI AG, Belgium) to stop bleeding. Prior to release, the bats were administered with a subcutaneous dose of Ringerʼs lactate solution and 5% glucose to replace energy and fluids. All bats were released at the site of capture and/or rescue within one hour of capture for blood sampling, while mice remained alive in the laboratory.

An i-STAT portable clinical analyser for veterinary use (EC8+ diagnostic cartridge, Abaxis, Union City, CA, USA) was used to measure blood profile parameters based on electrochemical sensing technologies, i.e. haematocrit (Hct, L/L), haemoglobin (Hb, g/L), sodium (Na, mmol/L), potassium (K, mmol/L), chloride (Cl, mmol/L), blood urea nitrogen (BUN, mmol/L), glucose (Glu, mmol/L) and total dissolved carbon dioxide (tCO2, mmol/L). Blood cell counts were enumerated using a Nihon Kohden MEK-5208 K cell counter (Nihon Kohden Corporation, Tokyo, Japan). A Romanowsky-stained blood smear was prepared to count white blood cell differential.

Respiratory burst response in both bats and laboratory mice was evaluated using luminol-enhanced chemiluminescence as a measure of phagocyte activity, as described previously [5, 75]. The reaction mixture contained blood diluted 1:50 in Hank’s balanced salt solution, luminol (Sigma-Aldrich Merck KGaA, Darmstadt, Germany) dissolved in borate buffer, and Zymosan A (Sigma-Aldrich Merck KGaA, Darmstadt, Germany). Zymosan A concentration in the reaction mixture was 0.25 mg/ml. Chemiluminescence kinetics were measured for two hours at two different temperatures using a Cytation 3 M reader (BioTek Instruments, Inc., Winooski, VT, USA). To reflect the body temperature of bats re-warming from daily torpor and mammals maintaining homeothermy, luminometric detection was performed at cell incubation temperatures of 25 °C for bats and 38 °C for laboratory mice, reflecting their physiological state based on body temperature measurements at the time of blood collection [24].

### Data analysis

Based on the chemiluminescence data, we calculated the following fundamental respiratory burst parameters: time-to-start of response (Tstart), time-to-peak response (Tpeak), time-to-end of response (Tend), peak intensity (Peak) and total capacity (Integral, I). The total capacity value was also adjusted to the white blood cell count (Adjusted Integral, AI). Normal distribution of variables was tested using the Kolmogorov-Smirnov and Shapiro-Wilk tests. Variables that were not normally distributed were log transformed and re-checked for normality. After transformation, all parameters were normally distributed with a statistical significance threshold set at 0.01 due to the low subsample size. The log-transformed datasets (Table S1) were used for all subsequent statistical analyses. Univariate ANOVA with *p*-value corrected by Bonferroni correction was used to test the differences between species with LSD (Fisher’s Least Significant Difference) post-hoc test identifying which pairs of species were statistically different. We defined the resultant clusters to the highest level of distinction using k-means clustering on pooled blood profile and phagocyte activity parameters. The expected number of clusters for each calculation was defined as the number of species (three), with missing parameter values being substituted by means.

The effect of blood profile parameters on phagocyte activity was estimated using multiple regressions, with both the response variables and explanatory variables log-transformed to achieve additivity of effects and to increase homogeneity of variance in the response variables. The predictive model was selected using stepwise selection with bidirectional elimination, using Akaike information criterion (AIC) at each step. The significance of a particular variable in the final model was checked by partial F-test with Bonferroni correction of *p*-value. Bidirectional elimination is essentially a forward selection procedure but with the possibility of deleting a selected variable at each step when there are correlations between variables, as in backward elimination [76]. Statistical analysis was performed in Statistica for Windows v. 13.3 or in R software using the support package mass [77].

## Supplementary information

**Additional file 1 Table S1.** Supporting information data (log-transformed) on blood parameters and phagocyte activity in *Myotis myotis*, *Nyctalus noctula* and *Mus musculus*.

## Data Availability

The datasets used and/or analysed during the current study are available from the corresponding author on reasonable request. Log-transformed data on blood parameters and phagocyte activity accompany this published article as additional file Table S1.

## Abbreviations

Adjusted Integral: total capacity respiratory burst adjusted to the white blood cell count
ANOVA: Analysis of variance
BALB/c: (Bagg and Albino) laboratory mice
BUN: Blood urea nitrogen
Cl: Chloride
Glu: Glucose
Hb: Haemoglobin
Hct: Haematocrit
Integral: Total capacity respiratory burst
K: Potassium
LSD test: Least significant difference test
Na: Sodium
Peak: Peak intensity respiratory burst
RNS: Reactive nitrogen species
ROS: Reactive oxygen species
tCO2: total dissolved carbon dioxide
Tend: Time-to-end respiratory burst
Tpeak: Time-to-peak respiratory burst
Tstart: Time-to-start respiratory burst
WBC: White blood cell count
