# Supplementary material for: Phagocyte activity reflects mammalian homeo- and hetero-thermic physiological states
Source: BMC Vet Res. 2020 Jul 6;16:232. doi: 10.1186/s12917-020-02450-z (PMC7339577; doi:10.1186/s12917-020-02450-z)
Supplement: Supplementary file 1 — Additional file 1 Table S1. Supporting information data (log-transformed) on blood parameters and phagocyte activity in Myotis myotis, Nyctalus noctula and Mus musculus. [file 12917_2020_2450_MOESM1_ESM.pdf]

Title

Phagocyte activity reflects mammalian homeo- and hetero-thermic physiological states

Authors

Jiri Pikula, Tomas Heger, Hana Bandouchova, Veronika Kovacova, Monika Nemcova, Ivana Papezikova, Vladimir Piacek, Renata Zajíčková, Jan Zukal

Supplementary Table S1

Data (log-transformed) on blood parameters and phagocyte activity in *Myotis myotis, Nyctalus noctula* and *Mus musculus*

Species	Na	K	Cl	tCO2	BUN	Glu	pH	pCO2	HCO3	AnGAP	RBC	WBC
M. myotis	2,179	0,875	2,045	1,415	1,324	1,021	0,861	0,859	1,382	1,380	1,007	0,643
M. myotis	2,190	0,934	2,072	1,362	1,362	0,863	0,865	0,739	1,340	1,362	1,144	0,643
M. myotis	2,179	0,886	2,064	1,279	1,375	0,839	0,864	0,688	1,262	1,380	1,085	0,748
M. myotis	2,152	0,839	2,033	1,279	1,220	0,732	0,855	0,823	1,250	1,362	1,093	0,681
M. myotis	2,170	0,863	2,061	1,301	1,276	0,944	0,860	0,751	1,262	1,342	1,052	0,204
M. myotis	2,170	0,857	2,041	1,398	1,303	0,996	0,864	0,804	1,378	1,342	1,479	0,447
M. myotis	2,167	0,924	2,037	1,380	1,530	0,820	0,858	0,861	1,344	1,380	0,980	0,833
M. myotis	2,170	0,892	2,061		1,408	0,875					1,145	0,857
M. myotis	2,173	0,914	2,049	1,398	1,369	0,826	0,869	0,710	1,369	1,342	1,104	0,447
M. myotis	2,185	0,886	2,086	1,204	1,447	0,763	0,858	0,692	1,164	1,380	1,032	0,944
M. myotis	2,179	0,919	2,076	1,322	1,320	0,845	0,863	0,732	1,292	1,301	0,966	0,903
N. noctula	2,176	0,954	2,090	1,322	1,316	0,813	0,861	0,762	1,297		0,702	1,049
N. noctula	2,190	0,892	2,093	1,342	1,342	0,556	0,861	0,788	1,320	1,255	0,966	0,833
N. noctula	2,155	0,863	2,064	1,255	1,140	0,740	0,861	0,716	1,238	1,230	0,857	0,833
N. noctula	2,188	0,919	2,090	1,398	1,207	0,380	0,861	0,839	1,365	1,230	0,835	0,881
N. noctula	2,188	0,863	2,076	1,568	1,188	0,531	0,862	0,994	1,539	0,845	0,763	0,964
N. noctula	2,185	0,851	2,093	1,322	1,215	0,568	0,860	0,772	1,290	1,230	0,946	0,881
N. noctula	2,188	0,863	2,079	1,398	1,090	0,447	0,861	0,837	1,365	1,255	0,748	0,778
N. noctula	2,182	0,881	2,093	1,380	1,193	0,462	0,857	0,880	1,342	1,146	0,903	0,833
N. noctula	2,176	0,857	2,083	1,380	1,049	0,462	0,861	0,827	1,350	1,146	0,699	0,924
N. noctula	2,173	0,845	2,086	1,342	1,029	0,785	0,858	0,842	1,312	1,146	0,905	0,964
N. noctula	2,173	0,799	2,086	1,301	0,973	0,813	0,863	0,708	1,267	1,176	0,922	0,643
N. noctula	2,170	0,778	2,076	1,279	0,987	0,724	0,857	0,800	1,255	1,230	0,845	1,033
M. musculus	2,146	0,613	2,017	1,415	0,380	1,097	0,865	0,782	1,382	1,204	0,872	0,716
M. musculus	2,152	0,724	2,045	1,362	0,748	0,987	0,862	0,787	1,326	1,176	0,846	0,914
M. musculus	2,152	0,756	2,057	1,322	0,799	1,029	0,863	0,744	1,305	1,114	0,836	0,716
M. musculus	2,155	0,732	2,049	1,398	0,462	0,973	0,862	0,835	1,373	1,114	0,848	0,633
M. musculus	2,155	0,756	2,049	1,380	0,672	1,053	0,861	0,820	1,348	1,146	0,883	0,716
M. musculus	2,161	0,633	2,053	1,398	0,531	0,924	0,862	0,819	1,364	1,114	0,836	0,568
M. musculus	2,158	0,643	2,053	1,447	0,322	1,000	0,861	0,891	1,418	1,000	0,829	0,519
M. musculus	2,164	0,716	2,068	1,342	0,785	1,045	0,855	0,873	1,310	1,146	0,836	0,716
M. musculus	2,146	0,806	2,045	1,322	0,778	0,964	0,862	0,754	1,294	1,176	0,881	0,785
M. musculus	2,155	0,732	2,053	1,362	0,708	1,000	0,860	0,806	1,326	1,146	0,782	0,491
M. musculus	2,161	0,748	2,057	1,342	0,699	0,964	0,858	0,825	1,310	1,204	0,809	0,633
M. musculus	2,152	0,724	2,041	1,380	0,643	1,009	0,858	0,871	1,348	1,176	0,862	0,230
M. musculus	2,164	0,699	2,053	1,398	0,580	0,954	0,860	0,870	1,375	1,146	0,881	0,301

MCV	MCH	PLT	Hct	Hb	Peak	T_{peak}	T_{start}	Intergal	Adjusted Integral	T_{end}
2,265	1,830	3,089	1,667	2,236	0,680	3,589	2,878	4,565	0,922	4,066
2,255	1,760	2,817	1,795	2,301	0,841	3,706	3,157	4,512	0,869	3,939
2,265	1,817	2,754	1,748	2,301	1,251	3,625	2,688	5,064	1,316	4,113
2,255	1,792	2,905	1,751	2,283	1,102	3,680	2,918	4,864	1,183	4,038
2,265	1,833	2,763	1,713	2,283	0,468	3,826	3,578	3,934	0,730	3,866
2,255	1,742	2,772	1,803	2,292	0,572	3,654	3,063	4,462	1,015	4,048
2,292	1,848	2,584	1,667	2,225	1,120	3,653	2,830	4,914	1,081	4,071
2,236	1,784	2,787	1,781	2,326	1,164	3,668	2,916	4,858	1,000	3,981
2,255	1,787	2,784	1,754	2,334	1,228	3,693	2,867	5,001	1,554	4,065
2,246	1,835	2,806	1,678	2,265	1,593	3,697	2,731	5,315	1,370	4,046
2,265	1,872	3,135	1,627	2,236	1,690	3,574	2,419	5,442	1,538	4,106
2,225	2,155		1,326	2,255	1,814	3,668	2,537	5,572	1,523	4,105
2,246	1,971	2,912	1,606	2,334	1,114	3,748	3,073	4,809	0,976	3,991
2,236	2,000	2,930	1,494	2,255	0,999	3,732	3,115	4,676	0,843	3,964
2,236	2,041	2,934	1,465	2,274	1,509	3,754	2,837	5,278	1,397	4,086
2,236	2,149		1,401	2,310	1,290	3,790	2,959	5,126	1,163	4,135
2,236	1,998	2,986	1,580	2,342	1,448	3,773	2,940	5,151	1,270	4,024
2,246	2,089	2,932	1,394	2,236	1,549	3,794	2,878	5,328	1,550	4,100
2,236	1,991	2,820	1,531	2,292	1,063	3,792	3,186	4,714	0,882	3,967
2,225	2,139		1,326	2,236	1,850	3,821	2,500	5,923	1,998	
2,236	1,980	2,840	1,542	2,283	1,498	3,793	2,934	5,223	1,259	4,047
2,236	1,934	2,647	1,556	2,255	1,179	3,760	3,074	4,846	1,202	3,976
2,236	2,049	2,787	1,477	2,292	1,594	3,752	2,828	5,308	1,274	4,039
1,699	1,217	2,877	1,572	2,090	1,275	3,162	1,779	4,996	1,280	4,108
1,732	1,260	2,900	1,577	2,107	2,004	3,080	1,260	5,633	1,719	4,204
1,748	1,225	2,973	1,585	2,061	1,757	3,125	1,495	5,400	1,684	4,144
1,699	1,212	3,005	1,551	2,061	1,720	3,133	1,499	5,389	1,755	4,170
1,716	1,225		1,600	2,107	1,618	3,163	1,609	5,304	1,588	4,156
1,708	1,243	2,972	1,543	2,079	1,468	3,098	1,676	5,061	1,493	4,014
1,785	1,255	3,008	1,616	2,083	1,606	3,355	1,947	5,383	1,865	4,202
1,732	1,250	2,895	1,565	2,086	1,791	3,053	1,218	5,489	1,773	4,272
1,716	1,262	2,979	1,597	2,143	1,565	3,198	1,717	5,258	1,472	4,137
1,724	1,212	2,789	1,509	2,097	1,417	3,209	1,797	5,137	1,645	4,132
1,724	1,243	2,943	1,534	2,053	1,755	3,195	1,692	5,379	1,746	4,083
1,724	1,274	2,954	1,588	2,137	1,221	3,188	1,880	4,936	1,706	4,077
1,748	1,262	2,813	1,604	2,134	1,026	3,184	2,032	4,715	1,414	3,984
